# Timing Does Matter: Nerve-Mediated HDAC1 Paces the Temporal Expression of Morphogenic Genes During Axolotl Limb Regeneration

**DOI:** 10.3389/fcell.2021.641987

**Published:** 2021-05-10

**Authors:** Mu-Hui Wang, Chia-Lang Hsu, Cheng-Han Wu, Ling-Ling Chiou, Yi-Tzang Tsai, Hsuan-Shu Lee, Shau-Ping Lin

**Affiliations:** ^1^College of Bioresources and Agriculture, Institute of Biotechnology, National Taiwan University, Taipei, Taiwan; ^2^Department of Medical Research, National Taiwan University Hospital, Taipei, Taiwan; ^3^Graduate Institute of Oncology, National Taiwan University College of Medicine, Taipei, Taiwan; ^4^Graduate Institute of Medical Genomics and Proteomics, National Taiwan University College of Medicine, Taipei, Taiwan; ^5^Department of Internal Medicine, National Taiwan University Hospital and National Taiwan University College of Medicine, Taipei, Taiwan; ^6^Liver Disease Prevention and Treatment Research Foundation, Taipei, Taiwan; ^7^Agricultural Biotechnology Research Center, Academia Sinica, Taipei, Taiwan; ^8^Center of Systems Biology, National Taiwan University, Taipei, Taiwan; ^9^The Research Center of Developmental Biology and Regenerative Medicine, National Taiwan University, Taipei, Taiwan

**Keywords:** histone deacetylase, axolotl limb regeneration, blastema, stage-dependent gene modulation, wound epidermis

## Abstract

Sophisticated axolotl limb regeneration is a highly orchestrated process that requires highly regulated gene expression and epigenetic modification patterns at precise positions and timings. We previously demonstrated two waves of post-amputation expression of a nerve-mediated repressive epigenetic modulator, histone deacetylase 1 (HDAC1), at the wound healing (3 days post-amputation; 3 dpa) and blastema formation (8 dpa onward) stages in juvenile axolotls. Limb regeneration was profoundly inhibited by local injection of an HDAC inhibitor, MS-275, at the amputation sites. To explore the transcriptional response of post-amputation axolotl limb regeneration in a tissue-specific and time course-dependent manner after MS-275 treatment, we performed transcriptome sequencing of the epidermis and soft tissue (ST) at 0, 3, and 8 dpa with and without MS-275 treatment. Gene Ontology (GO) enrichment analysis of each coregulated gene cluster revealed a complex array of functional pathways in both the epidermis and ST. In particular, HDAC activities were required to inhibit the premature elevation of genes related to tissue development, differentiation, and morphogenesis. Further validation by Q-PCR in independent animals demonstrated that the expression of 5 out of 6 development- and regeneration-relevant genes that should only be elevated at the blastema stage was indeed prematurely upregulated at the wound healing stage when HDAC1 activity was inhibited. WNT pathway-associated genes were also prematurely activated under HDAC1 inhibition. Applying a WNT inhibitor to MS-275-treated amputated limbs partially rescued HDAC1 inhibition, resulting in blastema formation defects. We propose that post-amputation HDAC1 expression is at least partially responsible for pacing the expression timing of morphogenic genes to facilitate proper limb regeneration.

## Introduction

Axolotls have a remarkable capacity to regenerate multi-tissue structures. Following limb amputation, the exposed wound is covered rapidly by a specialized epithelium derived from keratinocytes around the wound periphery (Chalkley, 1954; Ferris et al., 2010). Rather than the cells migrating across the wound surface, this sheet of epithelial tissue is propelled from behind as cells at the periphery take up water and expand in volume (Tanner et al., 2009). Underneath the wound epidermis, progenitor cells aggregate and lead to the formation of a unique structure called the blastema. The blastema is a combination of lineage-restricted and multipotent progenitors that gives rise to the internal structures of the newly regenerated limb (Kragl et al., 2009; Currie et al., 2016; McCusker et al., 2016; Fei et al., 2017). The correct interaction between the epidermis and the underlying ST is necessary for promoting blastema cell proliferation (Boilly and Albert, 1990) and stump tissue histolysis (Scheuing and Singer, 1957) and guiding blastema outgrowth (Thornton, 1960). Changes in cellular behaviors correlate with changes in transcription (Geraudie and Ferretti, 1998). The cell lineage- and regeneration stage-specific expression patterns of various morphogenesis genes are critical for successful limb regeneration (Monaghan et al., 2009, 2012; Campbell et al., 2011; Knapp et al., 2013; Stewart et al., 2013; Wu et al., 2013; Díaz-Castillo, 2017). These data suggest that regeneration in axolotls is a highly orchestrated stepwise process requiring precise transcriptional modulation.

Many changes during injury are associated with the control of epigenetic mechanisms that alter chromatin structure and the properties of proteins that function in transcriptional regulation and cell signaling (Paksa and Rajagopal, 2017). One of these epigenetic mechanisms involves deacetylation of histone proteins by HDACs to make the regional chromatin structure more compact and has been revealed to be important in amphibian regeneration. HDACs have also been associated with promoting growth and proliferation (Glozak and Seto, 2007). Because of the lack of detailed genomic data until recently (Nowoshilow et al., 2018), few studies have examined epigenetic mechanisms during axolotl regeneration. A study on Xenopus limb bud regeneration showed that histone modifications are important for regulating genes that maintain intrinsic limb-cell identities (Hayashi et al., 2015). Pharmacological blockade of HDACs reduces HDAC activity and inhibits tail regeneration in Xenopus tadpoles and larvae axolotls (Taylor and Beck, 2012). Tseng et al. (2011) also used valproic acid (VPA) and trichostatin A (TSA) to inhibit Xenopus tadpole tail regeneration. The authors observed that *notch1* and *bmp2*, two developmentally regulated genes that are required for Xenopus tail regeneration, were aberrantly expressed upon TSA treatment, consistent with the idea that HDAC activity is required during regeneration to regulate gene expression. In our previous study (Wang et al., 2019), we observed biphasic expression of nerve-mediated HDACs during the wound healing and blastema formation stages of axolotl limb regeneration. Furthermore, we reproducibly demonstrated an obvious reduction in cell proliferation and a prominent absence of limb regeneration upon treatment with two HDAC inhibitors, MS-275 and TSA, suggesting the necessity of HDAC for successful limb regeneration after amputation.

To elucidate the potential mechanism underlying the roles of HDACs in amphibian appendage regeneration, we took advantage of our axolotl limb regeneration model to analyze the alteration of the transcriptome at the wound healing and blastema formation stages when HDAC activity was inhibited by MS-275. We performed unsupervised clustering of genes exhibiting similar expression patterns during early limb regeneration in the epidermis and soft tissue (ST) with or without MS-275 treatment. Using gene set enrichment analysis (GSEA), we found that post-amputation elevation of HDAC expression is required for the correct gene expression timing. Notably, genes involved in tissue development, differentiation, and morphogenesis were prematurely enriched at the wound healing stage upon MS-275 treatment. Q-PCR and functional analysis of candidate genes and pathways in independent animals were performed to further validate the necessity of HDAC1 activity in preventing premature activation of regeneration stage-dependent gene expression.

## Results

### Transcriptome Profiling of the Epidermis and ST During Early Limb Regeneration Upon HDAC1 Inhibition

**FIGURE 1:**
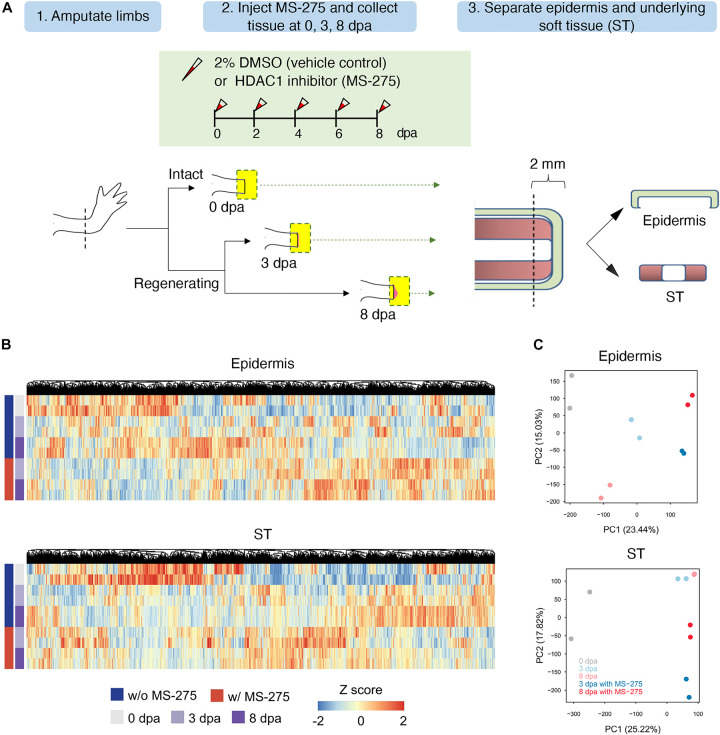
The effect of HDAC1 inhibition on the global transcriptome profiles of the epidermis and ST during the early stage of limb regeneration. **(A)** The HDAC1 inhibitor MS-275 was injected into the amputation site every other day after limb amputation to study the effect of HDAC1 depletion on transcriptome composition during early stage limb regeneration. Two biological replicates each of the epidermis and soft tissue (ST) at 0, 3, and 8 days post-amputation (dpa) were collected from HDAC1 inhibited and vehicle control animals. The epidermis and the underlying soft tissues were separated from the most distal part (2 mm) and then collected from 3 and 8 dpa, corresponding to the wound healing and blastema formation stages, respectively, and compared to the homeostatic control samples collected immediately after amputation. **(B)** Hierarchical clustering of overall transcripts based on the HDAC1 inhibitor MS-275, treatment and regeneration time course in the epidermis and ST. The expression values were transformed into z-scores for each gene, and a range of colors proportionate to the gene expression level are used (low: blue; high: red). See also supplementary Excel sheet 1. **(C)** Principal component analysis (PCA) of transcriptome profiles for the epidermis (top) and ST (bottom). The values in parentheses are the percentages of variance explained by each PC axis.

In our previous study, we demonstrated two waves of HDAC1 expression post-amputation corresponding to the wound healing stage and the duration of blastema formation. Inhibiting HDAC activity with the HDAC inhibitor MS-275 or TSA impaired blastema formation and subsequent limb regeneration ability (Wang et al., 2019; Supplementary Figure 1). To investigate further the transcriptional programming underlying the mechanism of the inhibition of regeneration by MS-275, we performed deep RNA sequencing during early axolotl limb regeneration using the Illumina sequencing platform. Gene expression of two types of tissues (epidermis and the underlying ST) treated with DMSO (as a control) or MS-275 at 0 dpa (homeostatic control), 3 dpa (wound healing stage), and 8 dpa (blastema formation stage of regeneration) was profiled (Figure 1A). Tissue samples from 4 limbs of two animals at each time point and for treatment condition were pooled as one biological replicate. In total, 40 limbs from 20 animals were used for the transcriptome analysis.

Because the axolotl genome was incomplete, we mapped sequencing reads to the axolotl transcriptome (Nowoshilow et al., 2018; Supplementary Figure 2A). An average of 78% of sequencing reads from each sample could be aligned to known axolotl transcripts (Supplementary Table 1). To obtain more functional information for each gene, we reannotated the axolotl transcripts by a basic local alignment search tool (BLAST) search against the UniProt database (Supplementary Figure 2A). There were 127,858 and 128,513 expressed transcripts identified in the epidermis and ST, respectively. Hierarchical clustering and principal component analysis (PCA) of the expression profiles for all samples revealed that the expression patterns depended primarily on regeneration status and MS-275 treatment (Figures 1B,C, and Supplementary Figure 2B). These data demonstrate that HDAC1 inhibitor treatment can generally be distinguished based on gene expression profiles.

### The Post-amputation Activity of HDAC Is Required for Correct Expression Timing of Genes Involved in Tissue Development, Differentiation, and Morphogenesis in ST

**FIGURE 2:**
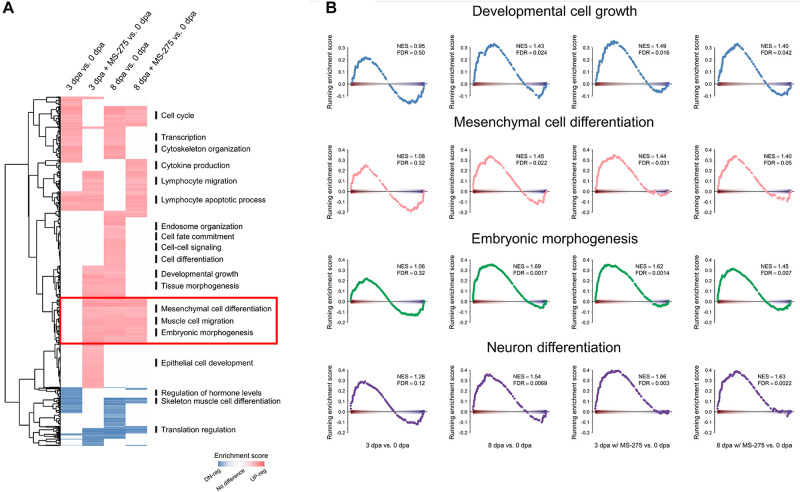
Premature enrichment of morphogenesis-related genes in the ST of the regenerating limb in the absence of HDAC1 activity. **(A)** The heatmap shows the results of GSEA at 3 dpa vs. 0 dpa or 8 dpa vs. 0 dpa with or without MS-275 treatment on gene sets defined by Gene Ontology. Red and blue indicate the gene sets significantly and positively (red) or negatively (blue) enriched, respectively, at 3 or 8 dpa (FDR < 0.05), and the intensity is associated with the normalized enrichment score (NES). **(B)** GSEA plots of the representative gene sets highlighted in **(A)**. NES, normalized enrichment score; FDR, false discovery rate.

To identify biological pathways important for critical stages of limb regeneration and those that were interfered with by HDAC inhibition, we grouped genes from each Gene Ontology (GO) term as a unit to perform Gene Set Enrichment Analysis (GSEA) with the following comparisons. As summarized in Figure 2A, we compared genes from each GO term for the wound healing stage (3 dpa; with or without MS-275 treatment) and homeostatic control (0 dpa) and for the blastema formation stage (8 dpa; with or without MS-275 treatment) and homeostatic control (0 dpa). There were 1,133 GO terms significantly enriched (FDR < 0.05) in at least one of the comparisons of each regeneration stage and treatment to homeostasis. As shown in Figure 2A, we used heatmaps to illustrate the activity of pathways and biological processes at different regeneration stages. We observed that the expression of genes related to transcriptional regulation and cytoskeletal organization was increased at both 3 and 8 dpa compared to 0 dpa, whereas these changes were not observed under HDAC1 inhibition (Figure 2A and Additional File A). These differences suggest that inhibition of HDAC1 affected active responses to amputation and signaling regulation for the normal early regeneration stages. The expression of genes associated with lymphocyte migration, on the other hand, was aberrantly upregulated by HDAC1 inhibition (Figure 2A and Additional File A). Notably, biological functions such as developmental growth and tissue morphogenesis, which were observed only at 8 dpa vs. 0 dpa under normal regeneration, were observed earlier at 3 dpa with MS-275 treatment vs. 0 dpa in the ST (Figure 2A and Additional File A). Thus, it seems that the inhibition of limb regeneration by blocking HDAC may be due to the right process occurring at the wrong time. In addition, many pathways associated with regeneration, such as developmental cell growth, mesenchymal cell differentiation, embryonic morphogenesis, and neuron differentiation, were enriched at both 8dpa and 8 dpa with MS-275 treatment vs. 0 dpa but were enriched earlier at 3 dpa with MS-275 treatment vs. 0 dpa (Figures 2A,B and Additional File A). Taken together, the post-amputation surge in HDAC activity is required for the correct expression timing of genes involved in tissue development, differentiation, and morphogenesis in the ST.

### Cluster Transition in the ST in Response to HDAC1 Inhibition

**FIGURE 3:**
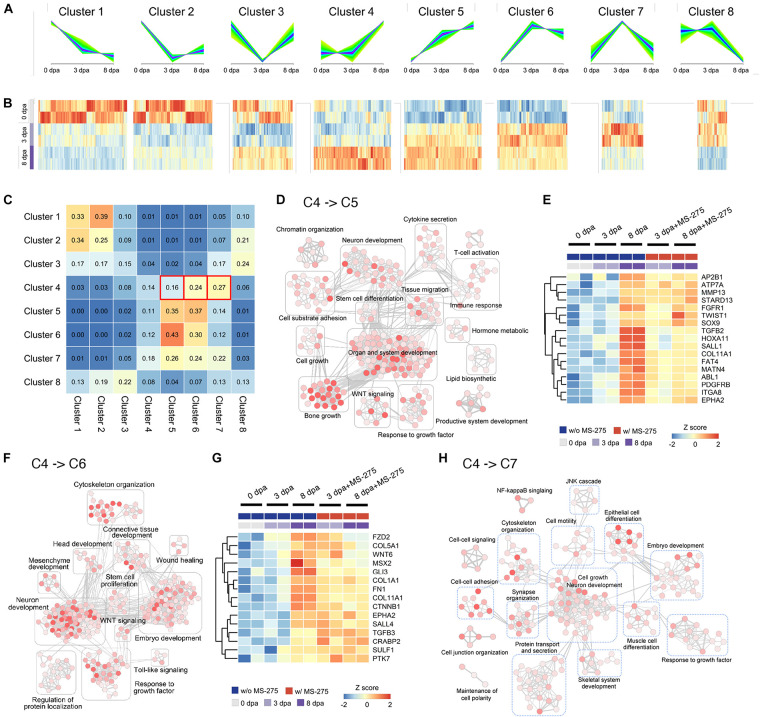
HDAC1 inhibition induced changes in regeneration stage-associated gene expression trends and the associated Gene Ontology terms based on unsupervised transcript clustering. **(A)** Unsupervised clustering of transcripts based on the trends in their expression patterns during normal regeneration at 0, 3, and 8 dpa. **(B)** The heatmap of the relative expression level of transcripts associated with each cluster. Transcript datasets from the ST in each stage without MS-275 treatment are shown for comparison. **(C)** The cluster transition matrix shows the proportion of genes in each given cluster (row) that exhibited changes in their expression pattern to those of other clusters (column) after MS-275 treatment. **(D)** Enrichment maps of gene sets significantly enriched (FDR < 0.05) by genes whose expression transitioned from cluster 4 to cluster 5 after MS-275 exposure. **(E)** The heatmap shows the expression values of selected genes whose expression transitioned from cluster 4 to cluster 5 after MS-275 exposure. **(F)** Enrichment maps of gene sets significantly enriched (FDR < 0.05) by genes whose expression transitions from cluster 4 to cluster 6 after MS-275 exposure. **(G)** The heatmap shows the expression values of selected genes whose expression transitioned from cluster 4 to cluster 6 after MS-275 exposure. **(H)** Enrichment maps of gene sets significantly enriched (FDR < 0.05) by genes whose expression transitions from cluster 4 to cluster 7 after MS-275 exposure. The red to white color gradient for each GO node indicates the significance of the enrichment for that particular GO term (red being more significant).

To obtain additional evidence that significant changes originally appeared at 8 dpa but occurred earlier at 3 dpa with MS-275 treatment, we divided genes into clusters based on the trend of their expression from 0 to 3–8 dpa. We first performed differential expression analysis at different time points and only considered the genes that showed significant differential expression (*p*-value < 0.05) in at least one of the time-point pairwise comparisons (namely, 0 vs. 3 dpa, 0 vs. 8 dpa, and 3 vs. 8 dpa) with DMSO or MS-275 treatment (Additional File B and Supplementary Table 2). A total of 37,798 genes were subjected to unsupervised clustering analysis by Mfuzz (Supplementary Table 3 and Additional File C) and were assigned to eight groups according to the temporal pattern of their expression (Figures 3A,B). We summarized the proportions (Figure 3C) and exact numbers of genes (Supplementary Table 3), and the gene identities (Additional file C) with stage-dependent expression patterns in the ST that switched among the defined clusters after MS-275 treatment.

We focused on the transitions from cluster 4 to 5 (C4 > 5) and cluster 4 to 6 (C4 > 6), as they may best represent the premature promotion of developmental and morphogenic relevant pathways. The expression pattern-based transition of genes from C4 > 5 was associated with development, including organ and system development (*notch2*, *col1a2*, *col2a1*, *rarg*, *hoxa10*, *hoxa11*, and *mmp9*), neuron development (*ntng1, sema3a, ngfr, fn1*, and *hoxd10*), bone growth (*col6a3, mmp13*, and *col12a1*), and the response to growth factors (*dkk3, itga8, tgfb2, msx2, twist1, fgfr1, pdgfrb*, and *sox9*) (Figures 3D,E). On the other hand, the expression pattern-based transition of genes from C4 > 6 was associated with development, including connective tissue development (*bmp1, ctnnb1, gli3*, and *hif1a*), embryo development (*col5a1, mmp2, sulf1, sall2, ptk7, tgfb2, dlx1*, and *sall4*), the response to growth factors (*col1a1* and *anos1*), neuron development (*cttn*, *eef2k*, *cthrc1*, *camsap2*, and *lama1*), and cytoskeleton organization (*col7a1*, *adamts12*, *mmp2*, and *gdf10*) (Figures 3F,G). Moreover, the expression pattern-based transition of genes from C4 > 7 was also related to development, such as neurogenesis (*hhip*, *bcl11b*, *nedd4l*, *bdnf*, *gdf5*, and *sox2*) and ECM structure organization (*itgab*, *nox1*, *tgfb2*, and *bmp7*) (Figure 3H and Additional File C).

In Figures 3E,G, we highlighted genes that exhibited cluster transition from C4 > 5 and C4 > 6, respectively. Several of these genes have been identified to be critical during the early stages of appendage regeneration. The expression of most of these regeneration-correlated regulators is thought to be significantly upregulated at the blastema formation stage at day 8 of normal regeneration (Monaghan et al., 2012; Currie et al., 2016). However, under HDAC1 inhibition, these genes are expressed too early, i.e., during the wound healing stage (*mmp13, fgfr1, twist1, sox9, tgfb2, pdgfrb, msx2, fn1, sall4*, and *tgfb3*). To validate the diverse gene expression patterns, we performed Q-PCR on independent animals (3 biological replicates for each condition; one biological replicate composed of 4 limbs from 2 animals; in total, 60 limbs from 30 animals were included in this validation study). Five out of the 6 genes tested exhibited similar premature elevation patterns when treated with MS-275, as indicated in the sequencing analysis (Supplementary Figure 3). Taken together, these results suggest that HDAC1 has an important role in preventing premature elevation of developmental genes post-amputation to ensure rigorous timing for ensuring successful blastema formation, which is critical for limb regeneration.

### Inhibiting HDAC1 Activity Changes the Expression Pattern of Cell Type-Associated Signature Genes

Intact limbs are composed of various cell types originating from lineages of the epidermis, endothelium, nerves, muscle and connective tissue (CT) (Rivera-Gonzalez and Morris, 2018). The compositions and ratios of multiple cell types may be key for successful limb regeneration. Differences in the cellular composition of the ST may be one of the possible reasons for failed limb blastema formation when post-amputation HDAC activity is blocked. Because of the lack of an available cell tracking system, we adopted a transcriptome-based approach to estimate the cell composition based on the expression level of the cell type-enriched genes defined by a previous publication (Leigh et al., 2018; Additional File D).

We explored the CT cell composition in the ST during regeneration because CT cells are the most abundant cells contributing to the blastema (Dunis and Namenwirth, 1977; Muneoka et al., 1986; Kragl et al., 2009; Currie et al., 2016). As illustrated in Supplementary Figure 4A, CT cells could be classified into two expression patterns groups after amputation based on their differential abundance across regeneration time points. The first group included fCT I, II, III, and IV cells, the numbers of which were decreased at 3 dpa, while the other group contained cycling cells, fCT V cells, periskeletal cells, and tendon cells, the numbers of which were increased at 8 dpa. Interestingly, except for genes representing cycling cells exhibiting delayed activation, the expression of genes representing various fCT cells, periskeletal cells and tendon cells was aberrantly upregulated or failed to be downregulated in the wound healing stage under MS-275 treatment (Supplementary Figure 4B). The deduced abnormal representations of cell compositions may be one of the causes of impaired limb regeneration. Furthermore, the delayed expression of genes representing cycling cells also hinted that the progress of regeneration was limited.

According to the results of cell composition analysis, the pattern transitions of fCT I, fCT II, fCT III, and fCT IV cells were similar to those of clusters 2 and 8 (Supplementary Figure 4C), and those of fCT V cells, periskeletal cells, and tendon cells were similar to those of clusters 4 and 6 (Figure 3F). Here, we focused on the transitions from cluster 2 to 8 (C2 > 8). Genes enriched in this transition of C2 > 8 were involved in various aspects of tissue remodeling, such as cellular component morphogenesis, cell junction organization, extracellular matrix (ECM) organization, and tissue migration, demonstrating that tissue repair and the promotion of positive matrix remodeling should have declined immediately upon the early stages of axolotl limb regeneration but that MS-275 treatment somehow caused a delay (Supplementary Figure 4C). While we cannot completely rule out the possibility that homeostatic tissues were slightly overrepresented in the sampling of MS-275-treated regeneration defective tissues, thus resulting in the higher transcript quantity of fCT-associated genes, the premature activation of periskeletal and tendon-associated genes could not be explained by the possible over-representation of homeostatic tissues. Moreover, based on the morphological observation (Supplementary Figure 1), overrepresentation of homeostatic tissue sampling may take place at 8 dpa but is less likely to occur at 3 dpa. These observations further support the main conclusion of premature elevation of blastema stage-expressing genes at the wound healing stage when the repressive histone modifier HDACs were inhibited.

### The Post-amputation Expression of HDAC Is Also Required for Correct Gene Expression Timing in the Epidermis

The initiation of blastema formation is dependent on the formation of the epidermis. Open wounds in the axolotl tail are rapidly closed by epidermal cells that migrate from the basal layer of the epidermis (Endo et al., 2004). Although there was no significant delay in wound healing between the control and MS-275 treatment in our limb regeneration model (Wang et al., 2019), inability of the right epidermal layers to cover the wound edge may still be an issue. To understand whether the composition of the epidermis may be affected by MS-275, cell composition analysis was performed on gene expression profiles of the epidermis using epidermal cell-related marker genes from a previous single-cell study (Nowoshilow et al., 2018; Supplementary Figure 5A and Additional File E). We found that expression in epidermal Langerhans, intermediate epidermis and small secretory cells was affected by HDAC1 inhibition, suggesting that the priority of cell migration might be changed (Supplementary Figure 5B).

To identify the genes associated with the potential changes in cell position, clustering analysis of 39,581 differentially expressed genes in at least one of the pairwise time points was performed, and these genes were classified into eight groups (Supplementary Figures 6A,B and Additional file F). Accordingly, the cluster transition from cluster 4 to 7 (C4 > 7) was consistent with expression in epidermal Langerhans, intermediate epidermis and small secretory cells (Supplementary Figures 5B, 6C, Supplementary Table 4, and Additional File G). Immune-related biological functions, including the inflammatory response, leukocyte-mediated immunity, leukocyte differentiation, and leukocyte migration, were enriched for genes that transitioned from C4 > 7 in the epidermis (Supplementary Figure 6D). Similarly, the GO terms associated with responses to wound and leukocyte activation and migration were only enriched at 8 dpa vs. 0 dpa under normal regeneration but were enriched earlier in the wound healing stage in MS-275-treated wound epidermis at 3 dpa vs. homeostatic epidermis at 0 dpa (Supplementary Figure 7 and Additional File H). Taken together, nerve-mediated HDAC1 activity is necessary for pacing gene expression at the right stage of regeneration in both the ST and wound epidermis.

### Wnt Inhibitor Partially Rescued MS-275-Induced Blastema Formation Defects

The current study identified many developmental relevant pathway-associated genes that were expressed earlier when HDAC1 activity was inhibited after amputation. Among them, we selected Wnt signaling pathway-associated genes to perform a proof of principle functional assay. Wnt signaling has been demonstrated to be involved in vertebrate limb regeneration; however, limb development requires spatial-temporal regulation of the Wnt signaling pathway (Yokoyama et al., 2011; Wischin et al., 2017). To examine whether premature elevation of the Wnt pathway is one of the causes of impaired regeneration upon HDAC1 inhibition, we used 15 independent juvenile axolotls and subjected their 30 limbs to 3 different treatments after amputation; control treatment, i.e., injection of vehicles; regeneration-inhibiting treatment, i.e., 25 mM MS-275 alone; and rescue treatment, i.e., 25 mM MS-275 plus 1 μM Wnt inhibitor. Limb regeneration was assessed every day until 26 dpa. A blastema could be easily seen at 8 dpa in the control group (Supplementary Figure 8B), whereas the MS-275 alone-treated axolotls displayed a lack of regeneration (Supplementary Figure 8C). The axolotls treated with MS-275 plus Wnt inhibitor exhibited a smaller blastema than the control axolotls (Supplementary Figure 8D). All control animals regenerated limbs with digits. Out of the 10 MS-275-treated limbs, 9 did not regenerate, and only 1 limb developed into the early bud blastema stage. In the MS-275 plus Wnt inhibitor group, 1 limb failed to regenerate, 8 developed into the blastema stage, and 1 limb further regenerated into the early differentiation stage. It is apparent that the addition of a Wnt inhibitor partially rescued the MS-275-induced blastema formation defects. Optimization of a regeneration stage-dependent Wnt inhibition protocol under HDAC1 inhibited amputated limb would be necessary to evaluate the full rescue effect. This experimental model can be applicable for functionally test the regeneration stage sensitive pathways identified in this study.

## Discussion

Regeneration in axolotls is initiated after wounding and depends on nerve-derived trophic factors (Vieira et al., 2019). The wound epidermis rapidly migrates and covers the wound within hours. In the next days, nerve fibers originating from the amputation plane innervate the wound epidermis, and the signaling loops between the nerve and wound epidermis build a signaling center called the apical epithelial cap (AEC). The AEC produces various signals that promote blastema cell dedifferentiation and proliferation (Makanae et al., 2013). The wound epidermis is a specialized epithelium required for the initiation of blastema formation and limb regeneration (Goss, 1956; Thornton, 1957, 1958; Mescher, 1976; Tassava and Garling, 1979), while its roles during the early stages of limb regeneration and the nerve-mediated HDAC molecular mechanisms mediating its transition remain largely unknown. In previous studies (Hay and Fishman, 1961; Satoh et al., 2012), the epidermis in the region proximal to the wound site remained proliferative after amputation, although the proliferation rate was reduced rate compared to that of the wound epidermis. Moreover, the initiating epidermal cells that rapidly cover the wound surface are derived from the basal epidermis (Endo et al., 2004; Ferris et al., 2010). We observed premature elevation of the expression of various cell type-associated signature genes when HDAC activity was inhibited (Supplementary Figure 5). Further validation by *in situ* characterization of marker genes representing the basal epidermis [collagen type xvii alpha 1 chain (col17a1)], proliferating epidermal cells (high levels of proliferating cell nuclear antigen (pcna), intermediate epidermis (krt12), and small secretory cells [SSCs, otogelin (otog)], is needed in the future to demonstrate clearly the HDAC1-associated cell composition changes in regenerating tissues.

The evolutionary loss of complete scar-free regenerative potential for multiple tissue types in mammalian and amphibian models coincides with the development of the immune system (Mescher and Neff, 2005). A successful allograft requires lower rejection (Kragl et al., 2009; McCusker and Gardiner, 2013; Nacu et al., 2013). A significantly higher success rate for axolotl allotransplantation is also associated with a lower immune response. It seems that the occurrence of an immune response suppresses regeneration. Although a strong immune response and regeneration seem to contradict each other, the presence of immune cells is required for regeneration. For instance, regeneration is inhibited when macrophage numbers are low, whereas healthy regeneration occurs when macrophages are present in sufficient numbers (Godwin et al., 2013, 2017). Furthermore, we also found a significant transition of the deduced epidermal cell population (Supplementary Figure 5B), and genes associated with immunity-related biological functions were expressed much earlier in the wound healing stage after MS-275 treatment (Supplementary Figure 6D). Differences in immune responses can tip the balance between scarring and regeneration (Godwin et al., 2013).

Blastema formation requires not only a specialized wound epithelium but also the coordination of the proliferation and migration of progenitors derived from muscle, bone, dermal fibroblasts, CT and hitherto undiscovered populations (Muneoka et al., 1986; Kragl et al., 2009; Currie et al., 2016; McCusker et al., 2016; Fei et al., 2017; Nowoshilow et al., 2018). CT contains key cell types for deciphering the molecular program of regeneration, as they express factors that guide the regeneration of appropriate limb parts (Carlson, 1975; Pescitelli and Stocum, 1980; Kragl et al., 2009; Nacu et al., 2013). We found that the expression levels of genes associated with mature fibrous CT (fCT) populations (fCT I, fCT II, fCT III, and fCT IV) were decreased after amputation (Figure 2A), which is consistent with a previous publication demonstrating the reduced representation of these cell types at the wound healing stage (Gerber et al., 2018). In contrast, cycling cells were enriched in the early stages of regeneration, and fCT V cells, periskeletal cells, and tendon cells were enriched around the blastema formation stages (Supplementary Figure 4). How CT cell compositions are modulated in the blastema has not been clarified because of the difficulty in isolation and deconstruction of blastema cells. Notably, signature genes associated with most CT cell types were highly enriched upon MS-275 treatment, except for cycling cells (Supplementary Figure 4). Because blastema progenitors are among early proliferating cells in stump tissues following amputation, we speculated that these cycling cells were inhibited by blocking HDAC1 activities. The reason for the failure of blastema formation may be changes in the activities of CT cells. This analysis revealed a general time-dependent progression of genes associated with different CT cell types. Future *in situ* staining or single-cell studies will be needed to trace the dynamic composition of each cell type.

Many candidate genes have been revealed to play essential roles in blastema initiation and maintenance based on their changes in gene expression patterns by bulk transcriptome studies from different stages of axolotl limb regeneration (Monaghan et al., 2009; Campbell et al., 2011; Knapp et al., 2013; Stewart et al., 2013; Wu et al., 2013; Voss et al., 2015; Bryant et al., 2017; Gerber et al., 2018). We found that MS-275 interfered with some regeneration-associated genes, which may have directly led to limb regeneration failure (Figures 3E,G). At the molecular level, the expression levels of extracellular matrix (ECM) deposition-related genes, including spalt-like transcription factor genes (*sall1* and *sall4*) (Stewart et al., 2013; Erickson et al., 2016; Li et al., 2021) and collagens (*col1a1*, *col5a1*, and *col11a1*), and ECM remodeling-related genes, including matrix metallopeptidase 13 (*mmp 13*) (Rayagiri et al., 2018), were low at the beginning of axolotl regeneration but increased at the blastema formation stage (Figures 3E,G). *Twist1* is an early marker of the limb blastema mesenchyme (Kragl et al., 2013). *Msx2* is used as a blastema marker gene (Satoh et al., 2007; Suzuki et al., 2005; Makanae et al., 2014). Sox9 is essential for sclerotome development and cartilage formation (Zeng et al., 2002). Notably, PDGFRB was identified as a chemotactic growth factor involved in wound healing or regeneration in an initial screen (Stewart et al., 2013) and was expressed in mesenchymal blastema by *in situ* hybridization (Currie et al., 2016). All of these genes that should not exhibit upregulation until the blastema stage were prematurely elevated at the wound healing stage when HDAC1 was inhibited.

Manipulating Wnt signaling activity at different stages of limb regeneration has different effects (Wischin et al., 2017). Chemical activation of Wnt signaling in the wound-healing stage inhibits limb regeneration. The treatment that is administered after the establishment of the blastema and before morphogenesis also causes disorganization of skeletal elements. In the current study, Wnt signaling was highlighted as one of the GO terms in the DEGs exhibiting cluster transition from 4 to 5 or 6 (Figures 3D,F), indicating that genes related to the Wnt pathway were activated earlier under the MS-275 treatment. We demonstrated partial rescue of blastema formation upon continuous inhibition of Wnt activity under HDAC1 inhibition (Supplementary Figure 8), indicating that aberrant Wnt activity was indeed one of the causes of MS-275 treatment-induced regeneration defects. Future profiling of Wnt pathway-related gene expression throughout the ∼26 days of limb regeneration in control and MS-275-treated limbs would pave the way to optimize a stage-specific, dosage-dependent protocol to further rescue HDAC1 inhibition-induced limb regeneration defects. Our preliminary results so far demonstrate the feasibility of performing limb regeneration rescue assays under HDAC1 inhibition conditions to study the novel pathway stage specifically modulated by post-amputation HDAC activities.

HDAC forms a complex containing distinct components that is believed to carry out different cellular functions, including regulation of the cell cycle (Rayman et al., 2002) and maintenance of stem cell pluripotency (Liang et al., 2008). HDACs control the correct timing of transcriptional programs among tissues and organs in the metamorphosis of anuran amphibians by interacting with thyroid hormone receptors (Shi, 2013). The recruitment of HDAC-containing corepressor complexes is critical for gene repression by unliganded thyroid hormone receptors in premetamorphic tadpoles and liganded thyroid hormone receptors that activate target gene transcription in metamorphosis. Such ligand switching behavior controls dramatic morphological development, and it is possible that regeneration-specific mechanisms may derepress HDAC corepressor complexes during regeneration to regulate transcription temporally. As HDAC1 is highly conserved in other vertebrates, examining whether it plays similar roles in other appendage regeneration models and whether it is differentially activated in non-regenerative systems after major limb injury may be worth exploring.

## Conclusion

Correct timing of regeneration-associated gene expression is one of the foundations of successful axolotl limb regeneration. Our study demonstrated the necessity of post-amputation HDAC activity in modulating regeneration stage-dependent gene expression activity. In the current proof of principle study, we demonstrated that inhibiting prematurely elevated Wnt pathway activity under treatment with the HDAC inhibitor MS-275 can partially rescue blastema formation ability. The HDAC1-mediated wound healing stage-specific repression of genes associated with tissue development, differentiation, and morphogenesis may be a prerequisite for blastema formation.

## Materials and Methods

### Animal Handling

Axolotls (*Ambystoma mexicanum*) were reared to juvenile age (12–16 cm snout to tail tip length) for all animal experiments. All the axolotls were kept in modified Holtfreter’s solution (118.4 mM NaCl, 1.3 mM KCl, 1.8 mM CaCl_2_, 1.6 mM MgSO_4_.7H_2_O). Prior to all experiments, the axolotls were anesthetized in 0.1% MS-222 (Sigma-Aldrich, St. Louis, MO, United States). Limb amputation was performed on the middle upper forelimbs on both the right and left sides, and tissues were harvested at 0, 3, and 8 dpa (Figure 1A). HDAC inhibitor injection into the amputated limbs of juvenile axolotls was performed as described by Wang et al. (2019). Briefly, 2 ml of 25 mM MS-275 (Selleckchem, Houston, TX, United States) was injected into the stumps immediately beneath the wound epidermis of juveniles every other day after amputation. The animal care and experimental procedures were approved by the Institutional Animal Care and Use Committee of National Taiwan University College of Medicine and were conducted in accordance with the approved guidelines.

### cDNA Library Preparation and Illumina Sequencing

Tissues from axolotls were subjected to total RNA extraction using TRIzol reagent (Invitrogen, CA, United States) according to the manufacturer’s instructions. RNA samples were purified using an RNeasy Mini Kit (Qiagen, Hilden, Germany) and then quantified on a Qubit 4 fluorometer (Invitrogen, CA, United States) and Qsep100 capillary gel electrophoresis (BiOptic, New Taipei City, Taiwan). All samples had an RNA quality number (RQN) of more than 9.0. To reduce variation among individuals within each group, tissue from both the right and left limbs of two animals at each time point was pooled together as one replicate. Two replicates from 4 animals (8 forelimbs) in total were prepared for one condition at each time point post-amputation. The 2 biological repeats for 5 conditions (3 dpa and 8 dpa with and without MS-275 treatment plus the homeostatic control at 0 dpa) includes tissues taken from 40 limbs of 20 axolotls. Ten RNA samples each from soft tissue (ST) and epidermis were subsequently used for cDNA library construction and Illumina deep sequencing.

Sequencing libraries were prepared using a TruSeq Stranded mRNA Preparation Kit (Illumina, CA, United States) according to the manufacturer’s instructions. Briefly, 10 μg of each total RNA sample was processed via poly-A selection with oligo(dT) magnetic beads and fragmentation. The resulting fragmented mRNAs were then subjected to first-strand cDNA synthesis using reverse transcription with random primers followed by second-strand cDNA synthesis using DNA polymerase I and RNase H (Invitrogen). Paired-end (PE) oligoadapters (Illumina) were then added to the cDNA fragments with T4 ligase. The resulting cDNA fragments were purified and enriched by polymerase chain reaction (PCR). The cDNA libraries were sequenced by the Illumina HiSeq2000 (Illumina) system, which generates PE raw reads approximately 150 bp in size.

### Transcriptome Reannotation

The axolotl transcriptome was obtained from https://axolotl-omics.org and annotated using two approaches: (i) the nucleotides were BLASTed (Altschul et al., 1990) against the UniProt database (BLASTx, *e*-value threshold 1e-5) and (ii) amino acid sequences were obtained based on the predicted open reading frames (ORFs) by TransDecoder and then BLASTed against the UniProt database (BLASTp, *e*-value threshold 1-e5). Various organism protein names were mapped to human gene symbols via the R package biomaRt (Durinck et al., 2005).

### Processing and Analysis of RNA-Seq Data

The raw FASTQ files were checked with FastQC (v0.11.7) and trimmed with cutadapt (v2.10). The qualified reads were aligned to the axolotl transcriptome using bowtie2 (v.2.3.4) (Langmead and Salzberg, 2012). The expression of each transcript was quantified using RSEM (Li and Dewey, 2011) and presented as log_2_TPM (transcript per million) for further analyses. Normalization across all samples was performed by the trimmed mean of the M-values (TMM) method implemented in the edgeR package (McCarthy et al., 2012). Differential expression analysis was performed using the limma package (Ritchie et al., 2015). First, linear models were constructed based on gene expression profiles and the experimental conditions, including time point and MS-275 treatment, utilizing the lmFit function. Second, the contrasts.fit function was employed to compute estimated coefficients and standard errors for a given experimental comparison. Finally, an empirical Bayes framework implemented in the eBayes function was used to compute the statistics of differential expression of all genes. The temporal clusters were determined by the fuzzy c-mean algorithm produced with the Mfuzz package (Futschik and Carlisle, 2005). For each tissue, genes with a *p*-value < 0.05 in one of the comparisons, i.e., 3 dpa vs. 0 dpa, 8 dpa vs. 0 dpa, dpa8 vs. dpa 3, 3 dpa w/MS-275 vs. 0 dpa, 8 dpa w/MS-275 vs. 0 dpa and 8 dpa w/MS-275 vs. 3 dpa w/MS-275, were considered for clustering analysis, and time-series profiles with and without treatment of MS-275 (namely, [0 dap, 3 dpa, and 8dpa] and [0 dpa, 3 dpa w/MS-275, and 8 dpa w/MS-275]) were merged into a single matrix for clustering analysis. To identify the optimal number of clusters, we performed repeated soft clustering for cluster numbers ranging from 4 to 27 to calculate the minimum centroid distance between two cluster centers produced by c-means clustering (Schwammle and Jensen, 2010).

### Function Analysis

The gene sets of GO biological processes were obtained from MSigDB (v7.0). Genes that do not exist in the axolotl transcriptome were identified and eliminated from the analysis. Overrepresentation analysis (ORA) and GSEA were performed using the functions implemented in the clusterProfiler package (Yu et al., 2012). For GSEA, the genes were ranked based on the log-transformed *p*-values derived from the limma test with signs set to positive/negative for a fold change of > 1 or < 1, respectively. An enrichment map was constructed using in-house R scripts (R statistical environment version 3.5.2.; Additional File I) and was visualized with Cytoscape.

### Cell Abundance Estimation

The transcriptomic markers of the different cell types were defined by previous studies (Gerber et al., 2018; Leigh et al., 2018; The gene lists are summarized in Additional Files 3, 4 for ST- and epidermis-associated cells, respectively). The estimated potential abundance of each specific cell type was estimated by the arithmetic mean of the normalized read counts from all signature genes.

### RT-Q-PCR for Validation

RNA was prepared using TRIzol Reagent (Invitrogen). RNA samples from the epidermis and ST were harvested at 0, 3, and 8 dpa for Q-PCR analysis. At the 0 dpa time point, the proximal 2 mm of the amputated parts were harvested immediately after amputation.

The epidermis and underlying soft tissue were separately collected. Total RNA from the collected tissues was extracted using TRIzol reagent (Invitrogen, Carlsbad, CA, United States). Reverse transcription using Superscript III reverse transcriptase (Invitrogen) was performed at 50°C. The first-strand cDNAs were diluted 10 times with nuclease free water and served as templates for Q-PCR. Reactions were performed in a total volume of 10 μl using the SYBR Green kit (Stratagene, La Jolla, CA, United States) with 0.8 μM of each primer following the manufacturer’s instructions. The sequences of the gene-specific primers (Supplementary Table 4) were determined based on our next-generation transcriptome sequencing data. Q-PCR was performed and analyzed with the ABI StepOne Real-Time PCR System with StepOne software version 2.1 (Applied Biosystems, Foster City, CA, United States).

### Local Injection of MS-275 and a Wnt Inhibitor

Among the biological/signaling pathways modulated by the post-amputation surge in HDAC1 activity, the Wnt signaling pathways is one of the pathways determined to be prematurely activated in the healing stage when amputated limbs are treated with MS-275. To examine whether the shift in Wnt signaling-associated gene expression from 8 to 3 dpa may be causative of blastema formation defects upon HDAC1 inhibition, we performed rescue experiments with Wnt inhibitors. Two microliters of 1 μM Wnt inhibitor (Calbiochem, Billerica, MA, United States) was injected simultaneously with MS-275, or 25 mM MS-275 only or vehicle only was administered. The timing of inhibitor injection into the amputated limbs of juvenile axolotls was determined according to a study by Wang et al. (2019); Figure 1 and Supplementary Figure 8.

## Data Availability Statement

RNA-seq data from this study were deposited in the Gene Expression Omnibus (GEO) under accession number GSE157716.

## Ethics Statement

The animal study was reviewed and approved by the IACUC, NTU.

## Author Contributions

M-HW, C-LH, C-HW, L-LC, H-SL, and S-PL conceived and designed the experiments. M-HW performed chemical inhibition and isolated RNA for transcriptome analysis. C-LH performed computations and developed related resources. M-HW, C-LH, Y-TT, H-SL, and S-PL interpreted the results. H-SL provided funding and supervision throughout the study. M-HW, C-LH, and S-PL wrote the manuscript. All authors were involved in periodic discussions, contributed to manuscript editing, and approved the final version before submission.

## Conflict of Interest

The authors declare that the research was conducted in the absence of any commercial or financial relationships that could be construed as a potential conflict of interest.

## Supplementary Material

Supplementary Figure 1 The absence of early bud blastema formation induced by MS-275.

Supplementary Figure 2 Comparative analysis of transcriptome profiles.

Supplementary Figure 3 Validation of transcripts in cluster transition by Q-PCR.

Supplementary Figure 4 The deduced HDAC1 inhibition-associated cell composition changes based on the expression patterns of signature genes of specific connective tissue (CT) cell types in the ST during early limb regeneration.

Supplementary Figure 5 The deduced possible cell composition shift in WE under MS-275 treatment during the early stage of limb regeneration.

Supplementary Figure 6 Highlighted GO categories for genes that undergo expression-pattern transition in response to MS-275 treatment in epidermis.

Supplementary Figure 7 Premature or aberrant enrichment of specific GO terms in epidermal tissue under MS-275 treatment.

Supplementary Figure 8 Partial rescue of MS-275-induced regeneration inhibition by a Wnt inhibitor.

Supplementary Table 1 Summary of the sequencing read alignment to the reference transcripts.

Supplementary Table 2 Numbers of differentially expressed genes from each comparison.

Supplementary Table 3 Matrix illustrating the number of genes exhibiting gene expression trajectory changes after MS-275 treatment (reference for Figure 3C).

Supplementary Table 4 Matrix illustrating the number of genes exhibiting gene expression trajectory changes after MS-275 treatment (reference for Supplementary Figure 6C).

Supplementary Table 5 Primer sequences for Q-PCR.

Additional File A Supplementary Matrix to Soft Tissue GSEA analysis based on each gene ontology (reference for Figure 2).

Additional File B Soft tissue DEGs between each regeneration stage/treatment vs. homeostatic control.

Additional File C Mfuzz cluster Transition in ST, including *P*-value and gene list for highlighted GO term from each cluster switch (Reference for Figure 3 and Supplementary Table 3).

Additional File D Signature genes for each connective tissue cell type.

Additional File E Signature genes for each epidermal cell types.

Additional File F Epidermis DEGs between each regeneration stage/treatment vs. homeostatic control.

Additional File G Mfuzz cluster Transition in epidermis, including *P*-value and gene list for highlighted GO term from each cluster switch (Reference for Supplementary Figures 6C,D and Supplementary Table 4).

Additional File H Supplementary Matrix to Epidermis GSEA analysis based on each gene ontology (reference for Supplementary Figure 7).

Additional File I The in-house developed scripts for the present study.
