# Supplementary material for: Timing Does Matter: Nerve-Mediated HDAC1 Paces the Temporal Expression of Morphogenic Genes During Axolotl Limb Regeneration
Source: Front Cell Dev Biol. 2021 May 10;9:641987. doi: 10.3389/fcell.2021.641987 (PMC8143519; doi:10.3389/fcell.2021.641987)
Supplement: Supplementary Figure 1 — The absence of early bud blastema formation induced by MS-275. [file Data_Sheet_1.pdf]

Supporting information

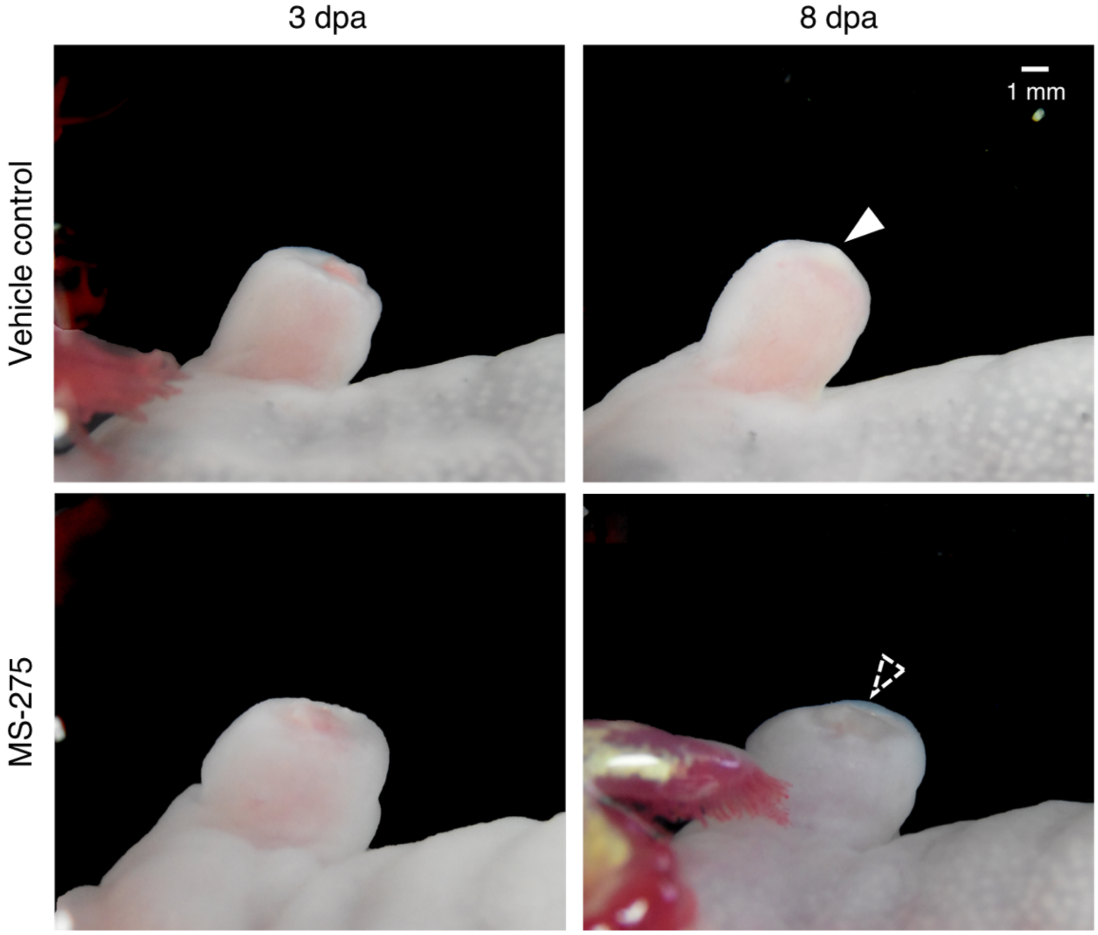

Supplementary Figure S1. The absence of early bud blastema formation induced by MS-275. Representative bright-field images showing the absence of blastema outgrowth at 8 dpa. The solid and dashed arrows indicate the blastema and the amputation plane, respectively. Scale bars: 1 mm.

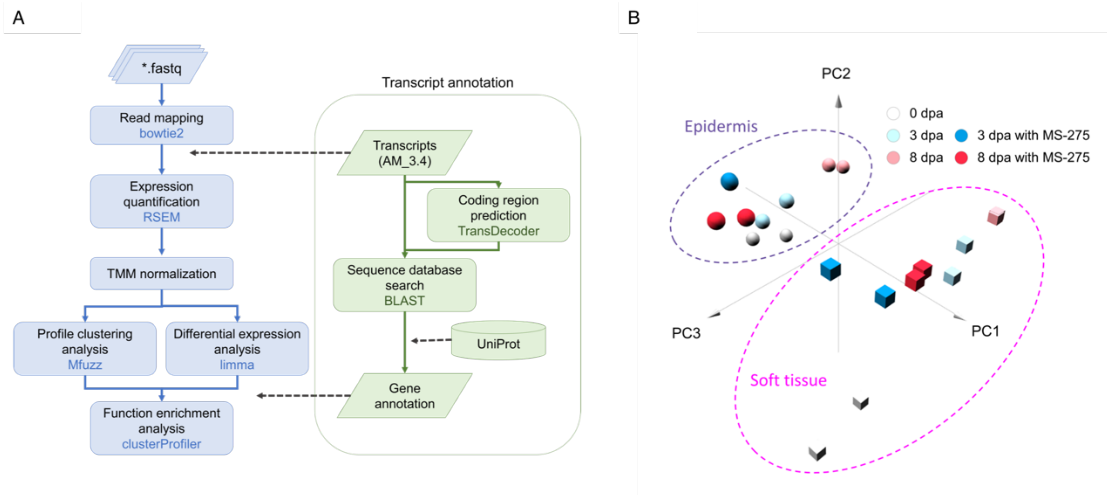

Supplementary Figure S2. Comparative analysis of transcriptome profiles. (A) Flowchart of the method used for the analysis of RNA-seq data and reannotation of the reference transcriptome of axolotl (Nowoshilow et al., 2018). (B) 3D projection plot of PCA of transcriptome profiles generated from this study.

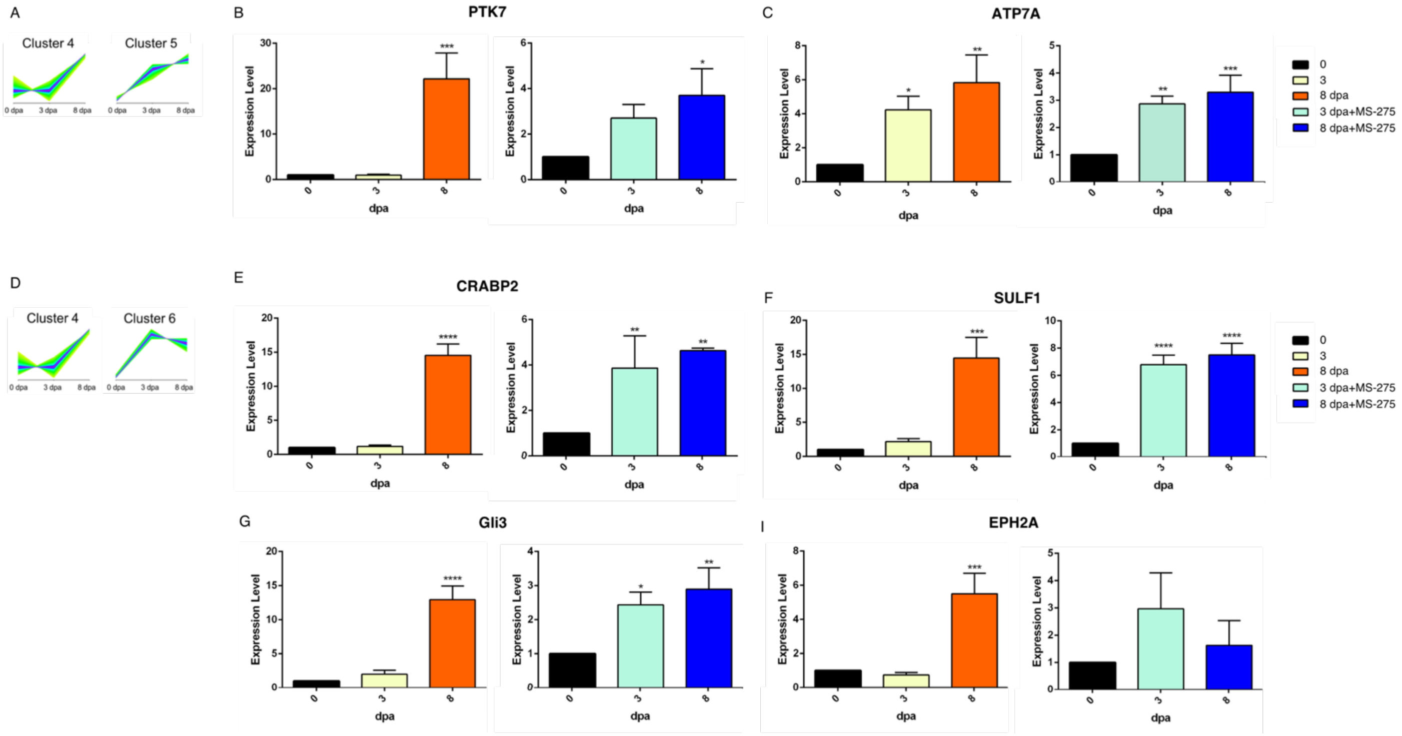

Supplementary Figure S3. Validation of transcripts in cluster transition by Q-PCR. (A) The trends in expression patterns from clusters 4 to 5 at 0, 3 and 8 dpa. (B and C) The expression of genes highlighted in Figure 3G was examined. (D) The trends in expression patterns from clusters 4 to 6 at 0, 3 and 8 dpa. (E-I) The expression of genes highlighted in Figure 3G was examined. The data are expressed as the means ± SEMs; n = 3 per group (3 biological repeats consisting of 4 arms from 2 animals per RNA sample; a total of 120 amputation/regeneration sites were studied). *P < 0.05.

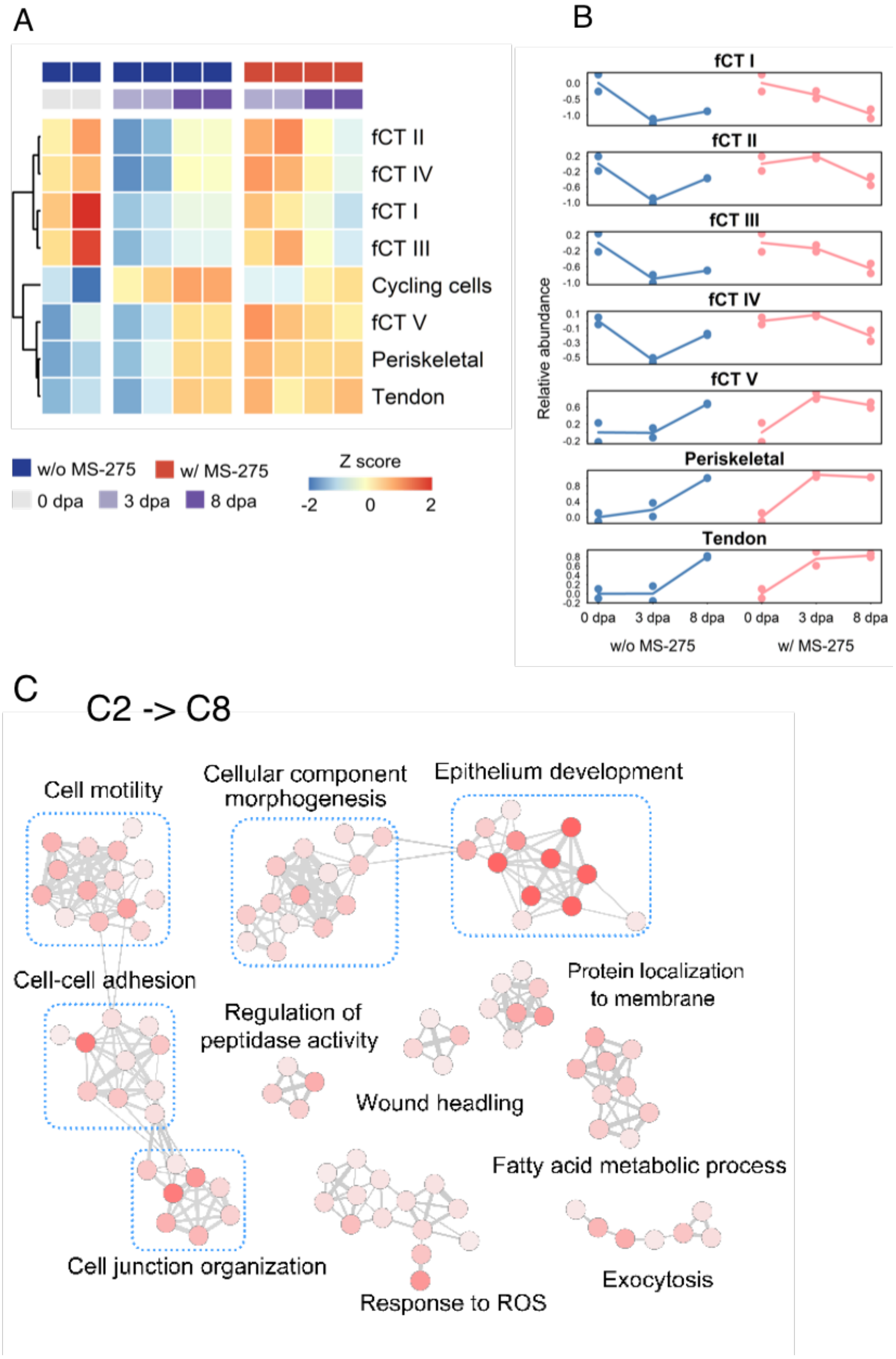

Supplementary Figure S4. The deduced HDAC1 inhibition-associated cell composition changes based on the expression patterns of signature genes of specific connective tissue (CT) cell types in the ST during early limb regeneration. (A) The heatmap shows the predicted relative abundance of various CT cells estimated

from the abundance of cell type-specific enriched signatures (red: high; blue: low). (B) MS-275 induced significant changes in the early regeneration stage-associated expression pattern of cell type-specific enriched gene cohorts. (C) Enrichment maps of gene sets significantly enriched (FDR < 0.05) by genes whose expression transitions from cluster 2 to cluster 8 after MS-275 exposure, exhibiting similar pattern changes observed from the cell type representative genes in fCT I, fCT II, fCT III, and fCT IV, as shown in (B). The red to white color gradient for each GO node indicates the significance of the enrichment for that particular GO term (red being more significant).

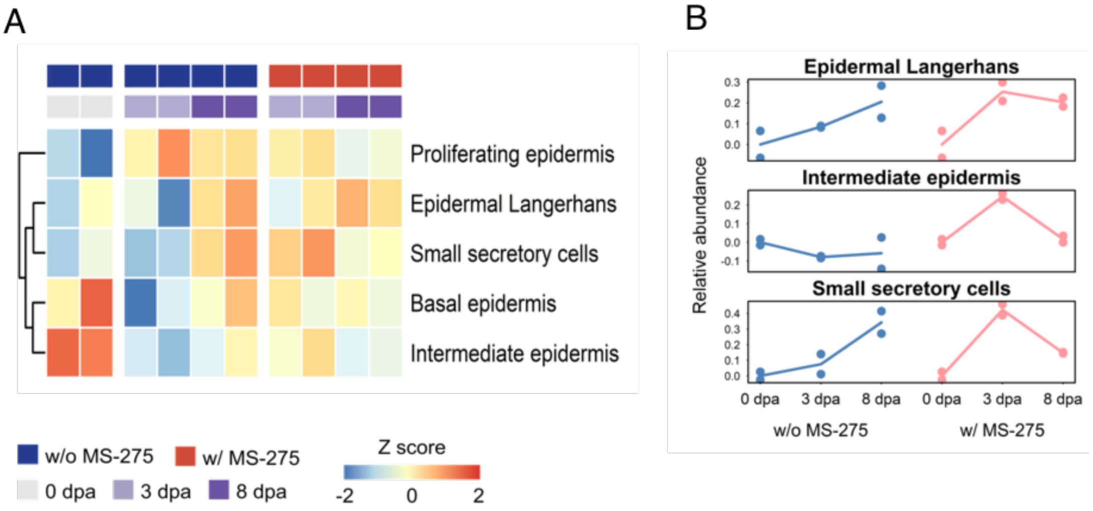

Supplementary Figure S5. The deduced possible cell composition shift in WE under MS-275 treatment during the early stage of limb regeneration. The putative abundance of epidermal cell types during WE exposure with or without MS-275 treatment. (A) The heatmap shows the predicted relative abundance of various epidermal cells estimated by the abundance of cell type-specific enriched signatures (red: high; blue: low). (B) The panel on the right demonstrates the premature enrichment of the deduced representation of epidermal Langerhans, intermediate epidermis and small secretory cells under MS-275 treatment.

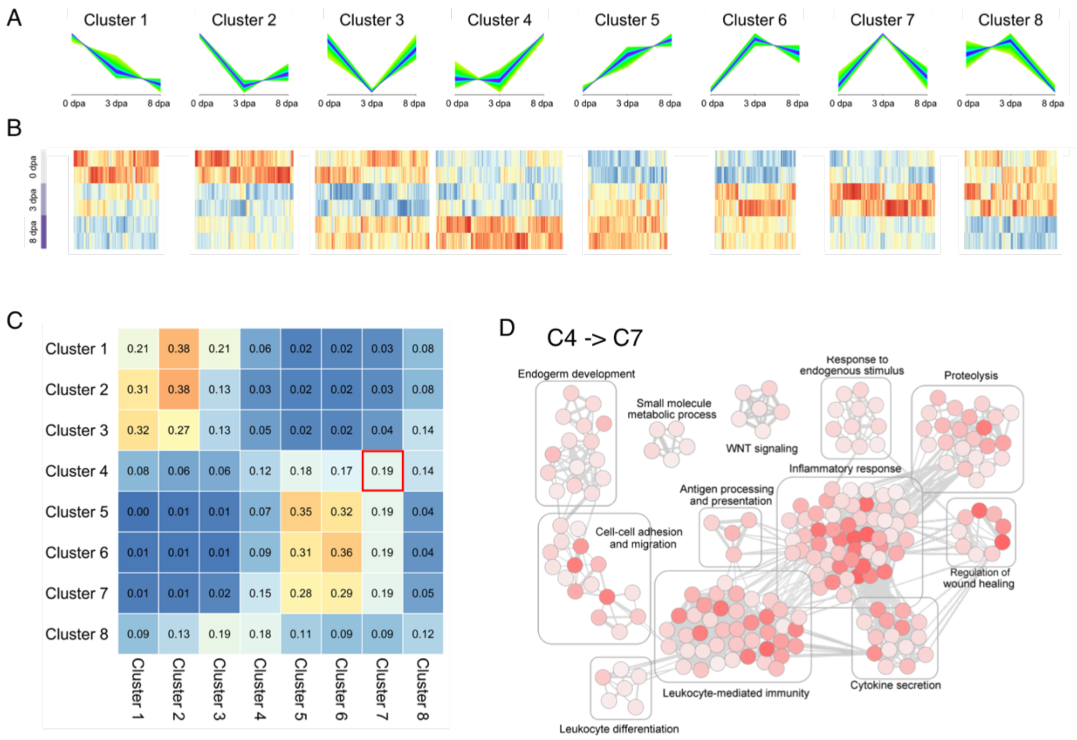

	Cluster 1	Cluster 2	Cluster 3	Cluster 4	Cluster 5	Cluster 6	Cluster 7	Cluster 8
Cluster 1	0.21	0.38	0.21	0.06	0.02	0.02	0.03	0.08
Cluster 2	0.31	0.38	0.13	0.03	0.02	0.02	0.03	0.08
Cluster 3	0.32	0.27	0.13	0.05	0.02	0.02	0.04	0.14
Cluster 4	0.08	0.06	0.06	0.12	0.18	0.17	0.19	0.14
Cluster 5	0.00	0.01	0.01	0.07	0.35	0.32	0.19	0.04
Cluster 6	0.01	0.01	0.01	0.09	0.31	0.36	0.19	0.04
Cluster 7	0.01	0.01	0.02	0.15	0.28	0.29	0.19	0.05
Cluster 8	0.09	0.13	0.19	0.18	0.11	0.09	0.09	0.12

Supplementary Figure S6. Highlighted GO categories for genes that undergo expression-pattern transition in response to MS-275 treatment in epidermis. (A) Unsupervised clustering of transcripts based on the trends in their expression patterns during normal regeneration at 0, 3 and 8 dpa. (B) The heatmap of the relative expression level of transcripts associated with each cluster. Transcript datasets from the epidermis of each stage without MS-275 treatment are shown for comparison. (C) The cluster transition matrix shows the proportion of genes in each given cluster (row) that exhibited changes in their expression pattern to those of other clusters (column) after MS-275 treatment. (D) Enrichment maps of gene sets significantly enriched (FDR < 0.05) by genes whose expression transitioned them from cluster 4 to cluster 7 after MS-275 exposure.

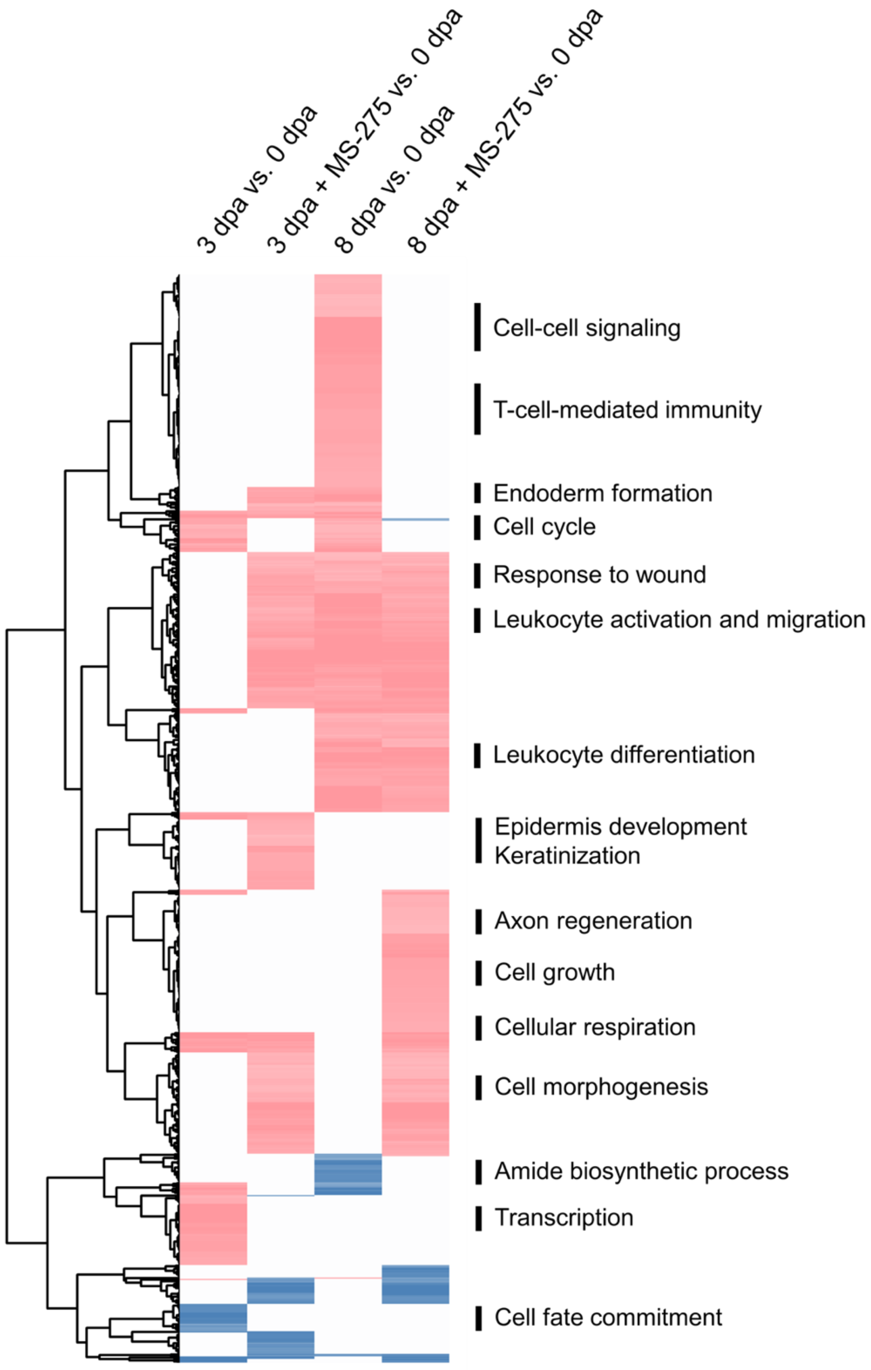

Supplementary Figure S7. Premature or aberrant enrichment of specific GO terms in epidermal tissue under MS-275 treatment. The heatmap shows the results of GSEA at 3 dpa vs. 0 dpa or 8 dpa vs. 0 dpa with or without MS-275 treatment on gene sets defined by Gene Ontology. Red and blue indicate the gene sets significantly positively (red) and negatively (blue) enriched, respectively, at 3 or 8 dpa (FDR < 0.05), and the intensity is associated with the normalized enrichment score (NES)

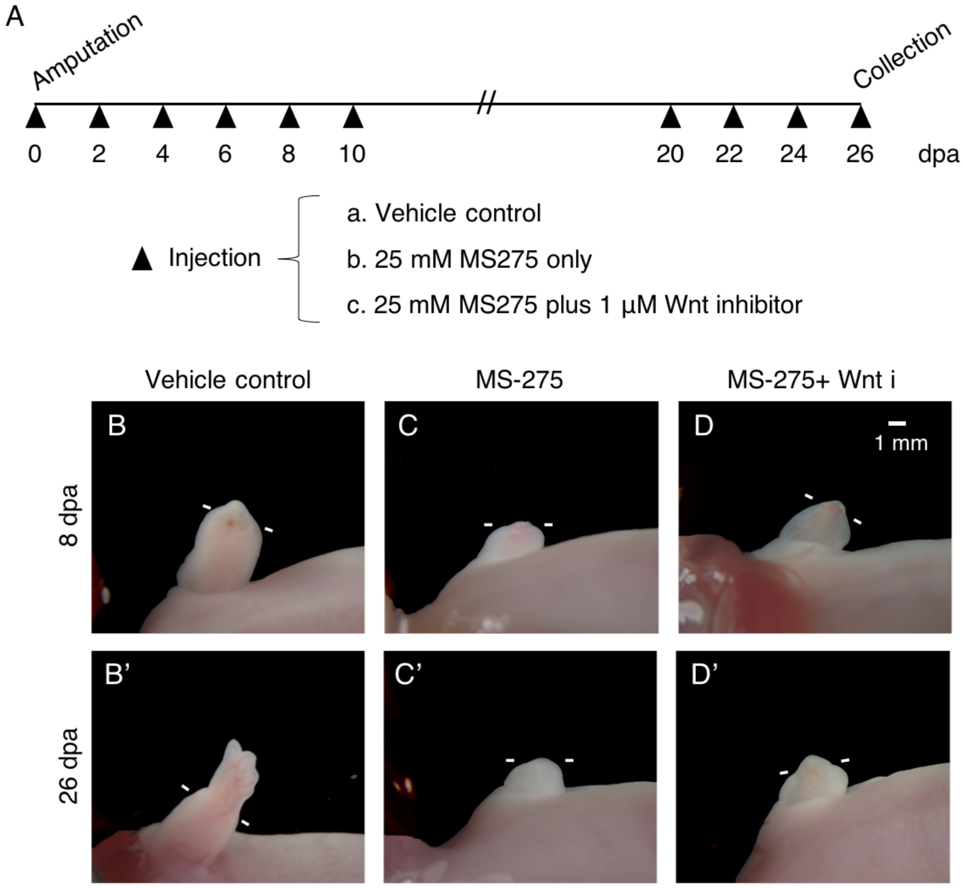

E

Treatment	Stage of regeneration		
	Non-regeneration	Blastema formation	Digit formation
Vehicle control	0	0	10
MS-275 only	9	1	0
MS-275 plus Wnt inhibitor	1	8	1

Supplementary Figure S8. Partial rescue of MS-275–induced regeneration inhibition by a Wnt inhibitor. (A) Experimental protocol indicating the timing of local injection (every other day) from amputation until 26 dpa. There were 3 injection groups: (a) the vehicle control, (b) 25 mM MS-275 alone, and (c) 25 mM MS-275 plus 1 μM Wnt inhibitor groups. Representative photos at 8 dpa showing blastema formation in group a (B), a lack of regeneration in group b (C), and a smaller blastema in group c (D). At 26 dpa, a new limb formed with four digits in group a (B'), there was no regeneration in group b (C'), and a blastema was observed in group c (D'). (E) A table showing the final outcomes for these 3 groups at 26 dpa. N=10 limbs in each group. The white bars indicate the amputation line. Scale bar= 1 mm.

Supplementary Table S1. Summary of the sequencing read alignment to the reference transcripts

Sample name	# of reads	Overall alignment rate
D3-ST-1	22,557,069	78.64%
D3-ST-2	36436803	80.59%
D3-ST-M1	19284219	76.49%
D3-ST-M2	28457400	77.32%
D3-WE-1	35204053	77.21%
D3-WE-2	40040745	76.47%
D3-WE-M1	38514305	77.14%
D3-WE-M2	34524802	75.96%
D8-ST-1	38603215	79.13%
D8-ST-2	30457341	79.43%
D8-ST-M1	32595534	78.19%
D8-ST-M2	42965033	80.49%
D8-WE-1	30721211	79.38%
D8-WE-2	35942941	78.26%
D8-WE-M1	46718700	77.71%
D8-WE-M2	34046014	78.87%
O-ST-1	46463921	78.69%
O-ST-2	27450973	80.87%
O-WE-1	41412254	88.23%
O-WE-2	35303186	78.49%

Supplementary Table S2. Numbers of differentially expressed genes from each comparison

	Epidermis	Soft tissue
3 dpa vs. 0 dpa	16,413	22,413
8 dpa vs. 0 dpa	16,677	24,892
8 dpa vs. 3 dpa	14,074	12,255
3 dpa w/MS-275 vs. 0 dpa	23,176	23,544
8 dpa w/MS-275 vs. 0 dpa	23,053	23,981
8 dpa w/MS-275 vs. 3 dpa w/MS-275	11,087	10,855

Supplementary Table S3. Matrix illustrating the number of genes exhibiting gene expression trajectory changes after MS-275 treatment (reference for Figure 3C,D,F,H).

Soft tissue

MS-275 treatment

Control	Cluster 1	Cluster 2	Cluster 3	Cluster 4	Cluster 5	Cluster 6	Cluster 7	Cluster 8
Cluster 1	1977	2425	661	69	43	84	316	669
Cluster 2	1872	1399	509	78	40	70	394	1257
Cluster 3	765	751	629	169	97	188	727	1038
Cluster 4	155	159	446	717	877	1360	1463	320
Cluster 5	5	21	108	590	1955	2045	727	65
Cluster 6	14	24	102	570	2192	1540	537	49
Cluster 7	31	42	154	554	868	766	630	104
Cluster 8	296	465	543	201	107	174	293	302

Supplementary Table S4. Matrix illustrating the number of genes exhibiting gene expression trajectory changes after MS-275 treatment (reference for Supplementary Figure S6C-D).

Epidermis

Control \ MS-275 treatment	Cluster 1	Cluster 2	Cluster 3	Cluster 4	Cluster 5	Cluster 6	Cluster 7	Cluster 8
Cluster 1	906	1639	912	245	77	108	118	354
Cluster 2	1540	1912	632	169	100	89	131	401
Cluster 3	1852	1531	714	277	139	138	255	802
Cluster 4	489	354	406	766	1155	1051	1163	887
Cluster 5	15	33	59	303	1445	1307	770	171
Cluster 6	22	25	37	356	1289	1487	791	159
Cluster 7	57	60	130	780	1473	1524	981	286
Cluster 8	427	599	905	863	509	425	425	556

Supplementary Table S5. Primer sequences for Q-PCR

Gene	Forward	Reverse
sulf1	GACGCACCAAGTTTGTCCAA	TGGTCACTTCATCTGCCGAA
atp7a	CAGCCGCTATAGGTACTCCAACTA	TTGTTGGATGTGGTGGCAAG
ptk7	GGGTCACTGTGTTTGCCAAT	CATGTCCTTGATGTCTGCGA
crabp2	AATCAGGCCAGCATTGTCCA	GCAGACTGAGTCCACATACAGGAA
gli3	CACTCTCCGATCACAGCTTTGA	TAAGTGACCGTATGACCCACTAGC
epha2	CGTAGACTATGGCACCAACTTC	CACCTCCACGTTTAGCTTCA
S21 ribosomal RNA*	ACTTGAAGTTTGTTGCCAGGAC	TGGCATCTTCTATGATCCCATC

*** served as an internal control.**

Lists of additional files

Additional file A Supplementary Matrix to Soft Tissue GSEA analysis based on each gene ontology (reference for Figure 2)

Additional file B. Soft tissue DEGs between each regeneration stage/treatment vs. homeostatic control.

Additional file C. Mfuzz cluster Transition in ST, including P value and gene list for highlighted GO term from each cluster switch (Reference for Figure 3 and Supplementary Table S3).

Additional file D. Signature genes enriched for each connective tissue cell type

Additional file E. Signature genes enriched for each epidermal cell types.

Additional file F. Epidermis DEGs between each regeneration stage/treatment vs. homeostatic control.

Additional file G. Mfuzz cluster Transition in epidermis, including P value and gene list for highlighted GO term from each cluster switch (Reference for Supplementary Figure S6C-D and Supplementary Table S4).

Additional file H Supplementary Matrix to Epidermis GSEA analysis based on each gene ontology (reference for Supplementary Figure S7)

Additional file I. The in-house developed scripts for the present study.
