# Supplementary material for: Timing Does Matter: Nerve-Mediated HDAC1 Paces the Temporal Expression of Morphogenic Genes During Axolotl Limb Regeneration
Source: Front Cell Dev Biol. 2021 May 10;9:641987. doi: 10.3389/fcell.2021.641987 (PMC8143519; doi:10.3389/fcell.2021.641987)
Supplement: Additional File I — The in-house developed scripts for the present study. [file Data_Sheet_10.PDF]

```
library(dplyr)
library(magrittr)

############################################################
#### Preprocessing and Normalization
############################################################
data.dir <- "/path/to/data"
countFile <- file.path(data.dir, "expected_count.txt")
sampleFile <- file.path(data.dir, "samples.txt")
geneFile <- file.path(data.dir, "GFZP01.1.annotation.updated.txt")

counts <- read.table(countFile, sep = "\t", header = TRUE, row.names = 1,
        stringsAsFactors = FALSE, check.names = FALSE)
counts <- round(counts)

samples <- read.table(sampleFile, sep = "\t", header = TRUE, row.names = 1,
        stringsAsFactors = FALSE, check.names = FALSE)

genes <- read.table(geneFile, sep = "\t", header = TRUE, row.names = "ID",
        stringsAsFactors = FALSE, check.names = FALSE, quote = "",
        comment.char = "")

edgeR.obj <- edgeR::DGEList(counts = counts, samples = samples, genes = genes,
remove.zeros = TRUE)
edgeR.obj <- edgeR::calcNormFactors(edgeR.obj)

obj.WE <- obj[, c("0-WE-2", "0-WE-3", "D3-WE-2", "D3-WE-3",
                  "D8-WE-1", "D8-WE-2", "D3-WE-M1", "D3-WE-M2",
                  "D8-WE-M1", "D8-WE-M2")]
obj.ST <- obj[, c("0-ST-2", "0-ST-4", "D3-ST-4", "D3-ST-5",
                  "D8-ST-1", "D8-ST-2", "D3-ST-M1", "D3-ST-M2",
                  "D8-ST-M1", "D8-ST-M2")]

keep.genes.WE <- rowSums(obj.WE$counts == 0) < 6
obj.WE <- obj.WE[keep.genes.WE,, keep.lib.sizes = FALSE]
obj.WE <- edgeR::calcNormFactors(obj.WE, method = "TMM")
keep.genes.ST <- rowSums(obj.ST$counts == 0) < 6
obj.ST <- obj.ST[keep.genes.ST,, keep.lib.sizes = FALSE]
```

```
obj.ST <- edgeR::calcNormFactors(obj.ST, method = "TMM")

saveRDS(obj.WE, file.path(data.dir, "WE.DEGList.rds"))
saveRDS(obj.ST, file.path(data.dir, "ST.DEGList.rds"))

### Heatmap of all profiled genes (Figure 1B)
annotation_color <- list(Day = c("grey90", "#9F9AC8", "#6B52A2"),
        Treatment = c(No = "#263C91", MS275 = "#D04A38"))

pheatmap::pheatmap(log2(edgeR::cpm(obj.WE)+1),
    breaks = seq(-2, 2, length.out = 100),
    cluster_rows = FALSE,
    show_colnames = FALSE,
    show_rownames = FALSE,
    annotation_row = subset(obj.WE$samples, select = c("Day", "Treatment")),
    treeheight_col = 12,
    legend = FALSE,
    annotation_legend = FALSE,
    annotation_names_row = FALSE,
    annotation_colors = annotation_color)

pheatmap::pheatmap(log2(edgeR::cpm(obj.ST)+1),
    breaks = seq(-2, 2, length.out = 100),
    cluster_rows = FALSE,
    show_colnames = FALSE,
    show_rownames = FALSE,
    annotation_row = subset(obj.ST$samples, select = c("Day", "Treatment")),
    treeheight_col = 12,
    legend = FALSE,
    annotation_legend = FALSE,
    annotation_names_row = FALSE,
    annotation_colors = annotation_color)

### PCA analysis (Figure 1C)
col.day <- c("WED0" = "grey70", "STD0" = "grey70",
         "WED3" = "#98CFE3", "STD3" = "#98CFE3",
         "WED8" = "#FF989F", "STD8" = "#FF989F",
         "WED3MS" = "#017AB3", "STD3MS" = "#017AB3",
```

```
        "WED8MS" = "#F61F35", "STD8MS" = "#F61F35")

pca <- prcomp(t(log2(edgeR::cpm(obj.WE)+1)), center = TRUE, scale. = TRUE)
eigs <- pca$sdev^2
pc.1 <- eigs[1] * 100 / sum(eigs)
pc.2 <- eigs[2] * 100 / sum(eigs)

xlab <- paste("PC", pc[1], " (", format(pc.1, digit = 2, nsmall = 2), "%)", sep
= "")
ylab <- paste("PC", pc[2], " (", format(pc.2, digit = 2, nsmall = 2), "%)", sep
= "")

par(mgp = c(1, 0.3, 0))
plot(pca$x[,c(1,2)], xlab = "", ylab = "", col = "white",
    xaxt = "n", yaxt = "n", tck = -0.01, main = "Epidermis")
points(pca$x[,c(1,2)], pch = 16, col = col.day[obj.WE$samples$Condition], cex =
16)

text(pca$x[,1], pca$x[,2]  + 1.5, labels = p.text, col = col, cex = 1.0)

axis(1, tck = -0.01, cex.axis = 0.6)
axis(2, las = 2, tck = -0.01, cex.axis = 0.6)

mtext(xlab, side = 1, line = 1.5, cex = 0.75)
mtext(ylab, side = 2, line = 1.5, cex = 0.75)
box()

pca <- prcomp(t(log2(edgeR::cpm(obj.ST)+1)), center = TRUE, scale. = TRUE)
eigs <- pca$sdev^2
pc.1 <- eigs[1] * 100 / sum(eigs)
pc.2 <- eigs[2] * 100 / sum(eigs)

xlab <- paste("PC", pc[1], " (", format(pc.1, digit = 2, nsmall = 2), "%)", sep
= "")
ylab <- paste("PC", pc[2], " (", format(pc.2, digit = 2, nsmall = 2), "%)", sep
= "")

par(mgp = c(1, 0.3, 0))
```

```
plot(pca$x[,c(1,2)], xlab = "", ylab = "", col = "white",
    xaxt = "n", yaxt = "n", tck = -0.01, main = "Soft tissue")
points(pca$x[,c(1,2)], pch = 16, col = col.day[obj.ST$samples$Condition], cex =
16)

text(pca$x[,1], pca$x[,2]  + 1.5, labels = p.text, col = col, cex = 1.0)

axis(1, tck = -0.01, cex.axis = 0.6)
axis(2, las = 2, tck = -0.01, cex.axis = 0.6)

mtext(xlab, side = 1, line = 1.5, cex = 0.75)
mtext(ylab, side = 2, line = 1.5, cex = 0.75)
box()

##############################################################
#### Differential expression analysis
##############################################################
group <- factor(obj.WE$samples$Condition)

## c: without MS-275; t: with MS-275
## 0, 3 and 8: 0 dpa, 3 dpa, and 8 dpa

design <- model.matrix(~0 + group)
colnames(design) <- gsub("group", "", colnames(design))
contrast.matrix <- limma::makeContrasts(
    c3.c0 = WED3-WED0,
    c8.c0 = WED8-WED0,
    c8.c3 = WED8-WED3,
    t3.c0 = WED3MS-WED0,
    t8.c0 = WED8MS-WED0,
    t8.t3 = WED8MS-WED3MS,
    t3.c3 = WED3MS-WED3,
    t8.c8 = WED8MS-WED8,
    levels = design)

v <- limma::voom(obj.WE, design)
vfit <- limma::lmFit(v, design)
vfit <- limma::contrasts.fit(vfit, contrasts = contrast.matrix)
```

```
efit <- limma::eBayes(vfit)
limma.res <- list()

for (tag in colnames(contrast.matrix)){
    limma.res[[tag]] <- subset(limma::topTable(efit, coef=tag, number = Inf),
            select = c("Transcript", "Symbol", "logFC", "P.Value", "adj.P.Val"))
}

saveRDS(limma.res, file = file.path(data.dir, "limma.res.WE.rds"))

## ST
group <- factor(obj.WE$samples$Condition)

design <- model.matrix(~0 + group)
colnames(design) <- gsub("group", "", colnames(design))
contrast.matrix <- limma::makeContrasts(
    c3.c0 = STD3-STD0,
    c8.c0 = STD8-STD0,
    c8.c3 = STD8-STD3,
    t3.c0 = STD3MS-STD0,
    t8.c0 = STD8MS-STD0,
    t8.t3 = STD8MS-STD3MS,
    t3.c3 = STD3MS-STD3,
    t8.c8 = STD8MS-STD8,
    levels = design)

v <- limma::voom(obj.ST, design)
vfit <- limma::lmFit(v, design)
vfit <- limma::contrasts.fit(vfit, contrasts = contrast.matrix)
efit <- limma::eBayes(vfit)
limma.res <- list()

for (tag in colnames(contrast.matrix)){
    limma.res[[tag]] <- subset(limma::topTable(efit, coef=tag, number = Inf),
            select = c("Transcript", "Symbol", "logFC", "P.Value", "adj.P.Val"))
}

saveRDS(limma.res, file = file.path(data.dir, "limma.res.ST.rds"))
```

```
############################################################
#### Identification of temporal-expression clusters
############################################################
## Averaging the expression value of two replicates
avgByGroup <- function(exp, group){
    out <- c()
    fs <- unique(group)
    for (f in fs){
        m <- apply(exp[,group == f], 1, mean)
        out <- cbind(out, m)
    }
    colnames(out) <- fs
    out
}

exprs.WE.avg <- avgByGroup(log2(edgeR::cpm(obj.WE)) + 1, obj.WE$Condition)
exprs.ST.avg <- avgByGroup(log2(edgeR::cpm(obj.ST)) + 1, obj.ST$Condition)

collectDiffGenes <- function(limma.obj, tags){
    genes <- c()
    for (tag in tags){
        dat <- limma.obj[[tag]]
        gl <- with(dat, rownames(dat[P.Value < 0.05,]))
        genes <- c(genes, gl)
    }
    genes <- unique(genes)
    genes
}

## Only condisering the differentially expressed genes
tags <- c("c3.c0", "c8.c3", "t3.c0", "t8.t3", "c8.c0", "t8.c0")

genes.WE <- file.path(data.dir, "limma.res.WT.rds") %>% readRDS() %>%
collectDiffGenes(tags)
genes.ST <- file.path(data.dir, "limma.res.ST.rds") %>% readRDS() %>%
collectDiffGenes(tags)
```

```
gene.df.WE.filter <- gene.df.WE[genes.WE,]
gene.df.ST.filter <- gene.df.WE[genes.ST,]

exprs.WE.filter <- exprs.WE.avg[genes.WE,]
exprs.ST.filter <- exprs.ST.avg[genes.ST,]

exprs.WE.Ctrl <- exprs.WE.filter[, c("WED0", "WED3", "WED8")]
exprs.WE.MS <- exprs.WE.filter[, c("WED0", "WED3MS", "WED8MS")]

exprs.ST.Ctrl <- exprs.ST.filter[, c("STD0", "STD3", "STD8")]
exprs.ST.MS <- exprs.ST.filter[, c("STD0", "STD3MS", "STD8MS")]

## Cluster analysis using Mfuzz
library(Mfuzz)

processESET <- function(exprs){
    eset <- new("ExpressionSet", exprs=exprs)
    eset.s <- standardise(eset)
    return(eset.s)
}

runMfuzz <- function(exprs, c=8){
    eset.s <- processESET(exprs)
    m1 <- mestimate(eset.s)
    cl <- mfuzz(eset.s, m=m1, c=c)
    return(cl)
}

if (TRUE){
    ## WE
    tmp1 <- exprs.WE.Ctrl
    row.names(tmp1) <- paste(row.names(tmp1), "ctrl", sep = "_")
    tmp2 <- exprs.WE.MS
    row.names(tmp2) <- paste(row.names(tmp2), "ms", sep = "_")

    tmp.WE <- rbind(tmp1, tmp2)

    mfobj.WE <- runMfuzz(tmp.WE, c = 8)
```

```
    saveRDS(mfobj.WE, file.path(data.dir, "WE.MFuzz.rds"))
    png(file.path(analysis.dir, "Mfuzz_WE.origin.png"), width = 32, height = 12,
units = "cm", res = 1200)
    par(mar = c(4, 4, 2, 2))
    mfuzz.plot2(processESET(tmp.WE), cl=mfobj.WE, time.labels=c("D0", "D3",
"D8"),
    x11 = FALSE, cex.lab = 1, cex.main = 1, cex.axis = 1,
    min.mem = 0.2, mfrow = c(2, 4))
    dev.off()

    ## ST
    tmp1 <- exprs.ST.Ctrl
    row.names(tmp1) <- paste(row.names(tmp1), "ctrl", sep = "_")
    tmp2 <- exprs.ST.MS
    row.names(tmp2) <- paste(row.names(tmp2), "ms", sep = "_")

    tmp.ST <- rbind(tmp1, tmp2)

    mfobj.ST <- runMfuzz(tmp.ST, c = 8)
    #mfuzz.plot(processESET(tmp.ST), cl=mfobj.ST, time.labels=c("D0", "D3",
"D8"),
    #   min.mem = 0.2, mfrow = c(4, 2))
    saveRDS(mfobj.ST, file.path(data.dir, "ST.MFuzz.rds"))
    png(file.path(analysis.dir, "Mfuzz_ST.origin.png"), width = 32, height = 12,
units = "cm", res = 1200)
    par(mar = c(4, 4, 2, 2))
    mfuzz.plot2(processESET(tmp.ST), cl=mfobj.ST, time.labels=c("D0", "D3",
"D8"),
    x11 = FALSE, cex.lab = 1, cex.main = 1, cex.axis = 1,
    min.mem = 0.2, mfrow = c(2, 4))
    dev.off()
}

## Re-name cluster numbers to make expression pattern consistency
if (TRUE){
    mfobj.WE <- readRDS(file.path(data.dir, "WE.MFuzz.rds"))
    order.WE <- c(1, 4, 2, 5, 8, 6, 3, 7)
    new.label.WE <- c(1, 3, 7, 2, 4, 6, 8, 5)
```

```
    mfobj.WE.edit <- mfobj.WE
    mfobj.WE.edit$size <- mfobj.WE.edit$size[order.WE]
    mfobj.WE.edit$membership <- mfobj.WE.edit$membership[, order.WE]
    colnames(mfobj.WE.edit$membership) <- c(1:8)
    cluster.WE <- factor(mfobj.WE$cluster)
    levels(cluster.WE) <- new.label.WE
    cluster.WE <- as.character(cluster.WE)
    cluster.WE <- as.numeric(cluster.WE)
    names(cluster.WE) <- names(mfobj.WE$cluster)
    mfobj.WE.edit$cluster <- cluster.WE
    saveRDS(mfobj.WE.edit, file.path(data.dir, "WE.MFuzz.modified.rds"))

    mfobj.ST <- readRDS(file.path(data.dir, "ST.MFuzz.rds"))
    order.ST <- c(2, 1, 8, 6, 5, 7, 3, 4)
    new.label.ST <- c(2, 1, 7, 8, 5, 4, 6, 3)
    mfobj.ST.edit <- mfobj.ST
    mfobj.ST.edit$size <- mfobj.WE.edit$size[order.ST]
    mfobj.ST.edit$membership <- mfobj.ST.edit$membership[, order.ST]
    colnames(mfobj.ST.edit$membership) <- c(1:8)
    cluster.ST <- factor(mfobj.ST$cluster)
    levels(cluster.ST) <- new.label.ST
    cluster.ST <- as.character(cluster.ST)
    cluster.ST <- as.numeric(cluster.ST)
    names(cluster.ST) <- names(mfobj.ST$cluster)
    mfobj.ST.edit$cluster <- cluster.ST
    saveRDS(mfobj.ST.edit, file.path(data.dir, "ST.MFuzz.modified.rds"))
}
```
